# Supplementary figures and images for: Impact of COVID-19 on patterns of drug utilization: A case study at national hospital
Source: PLoS One. 2024 Jan 19;19(1):e0297187. doi: 10.1371/journal.pone.0297187 (PMC10798442; doi:10.1371/journal.pone.0297187)

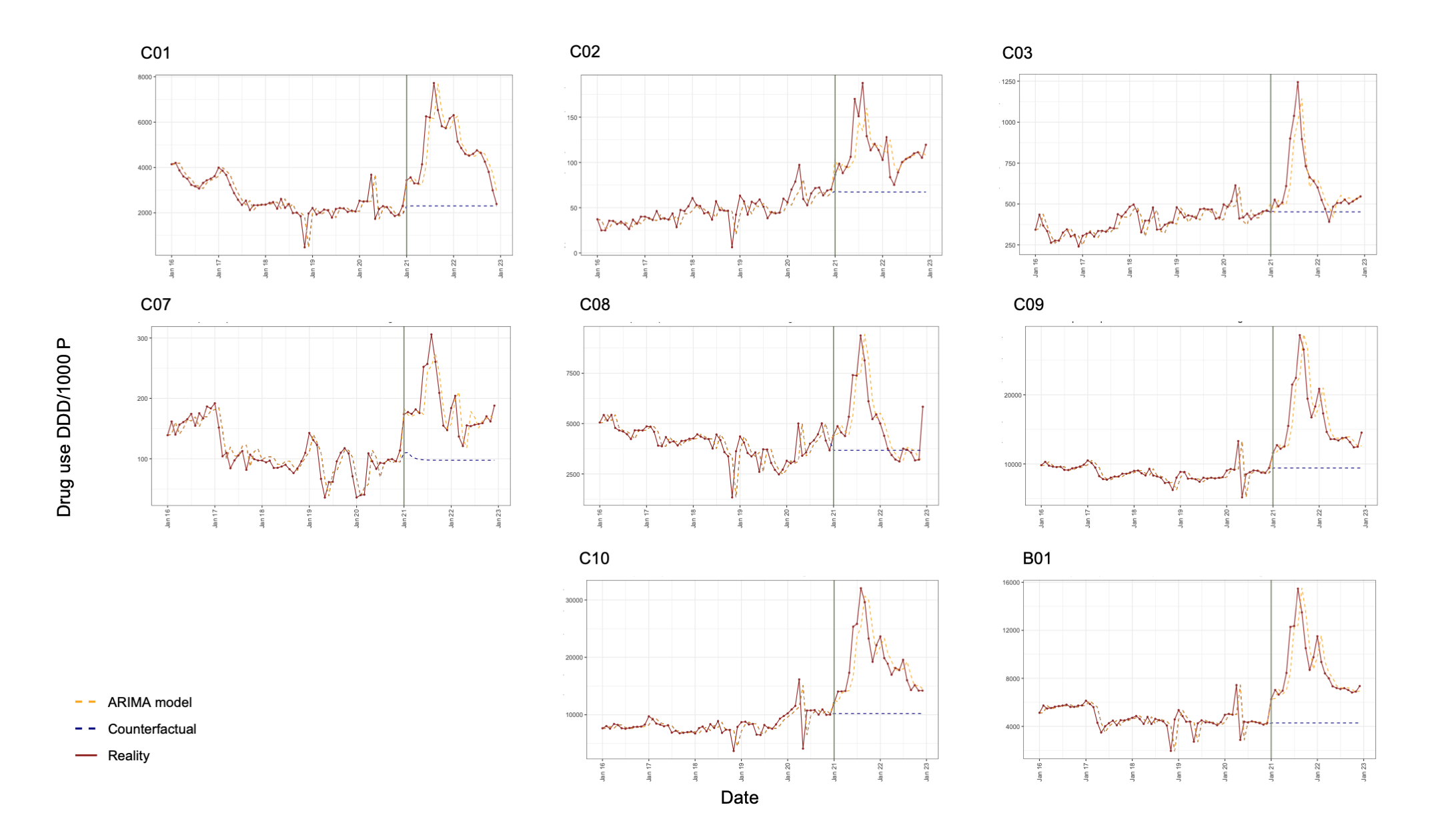

Supplement: S1 Fig — (TIF) [file pone.0297187.s001.tif]

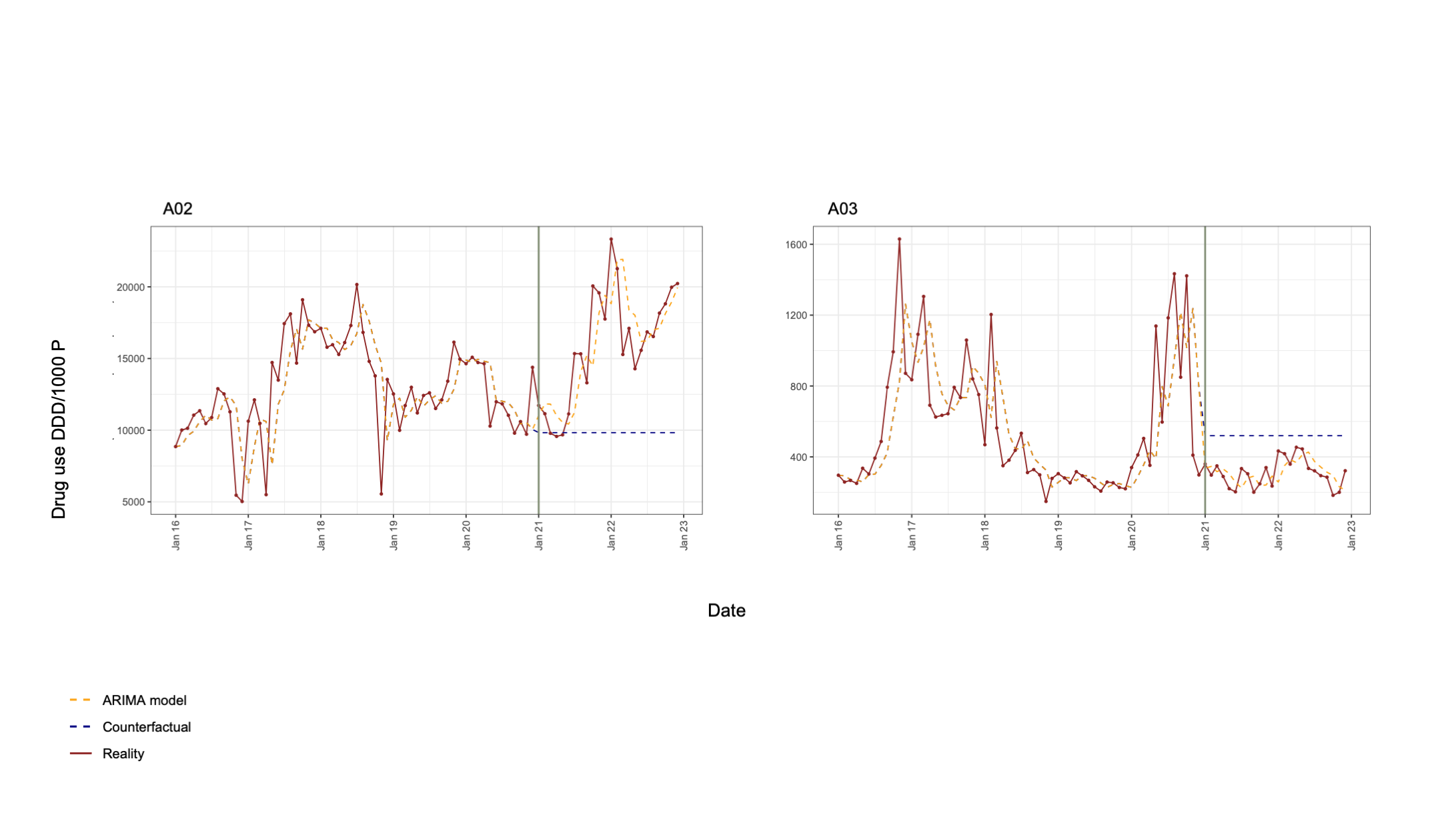

Supplement: S2 Fig — (TIF) [file pone.0297187.s002.tif]

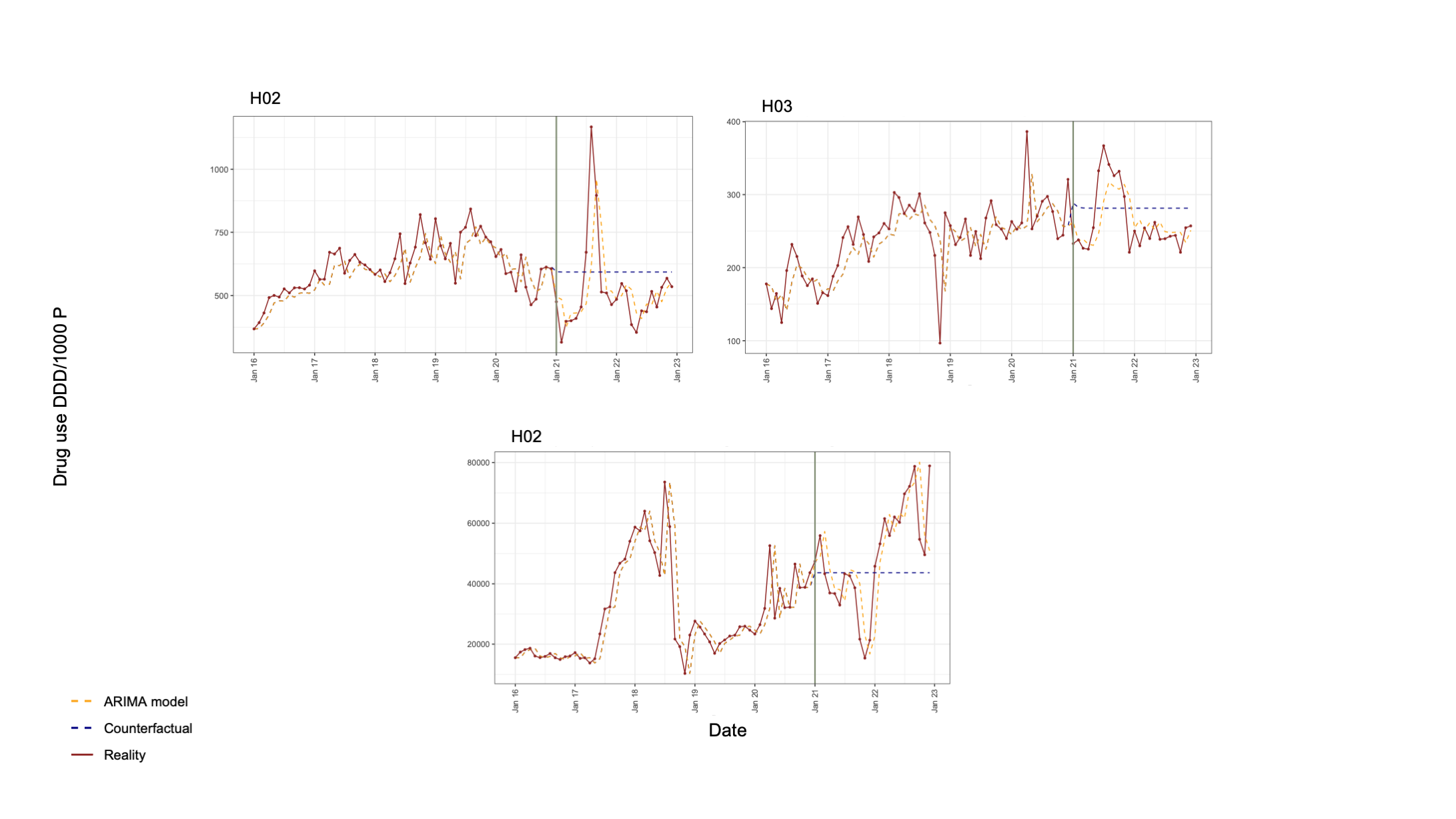

Supplement: S3 Fig — (TIF) [file pone.0297187.s003.tif]

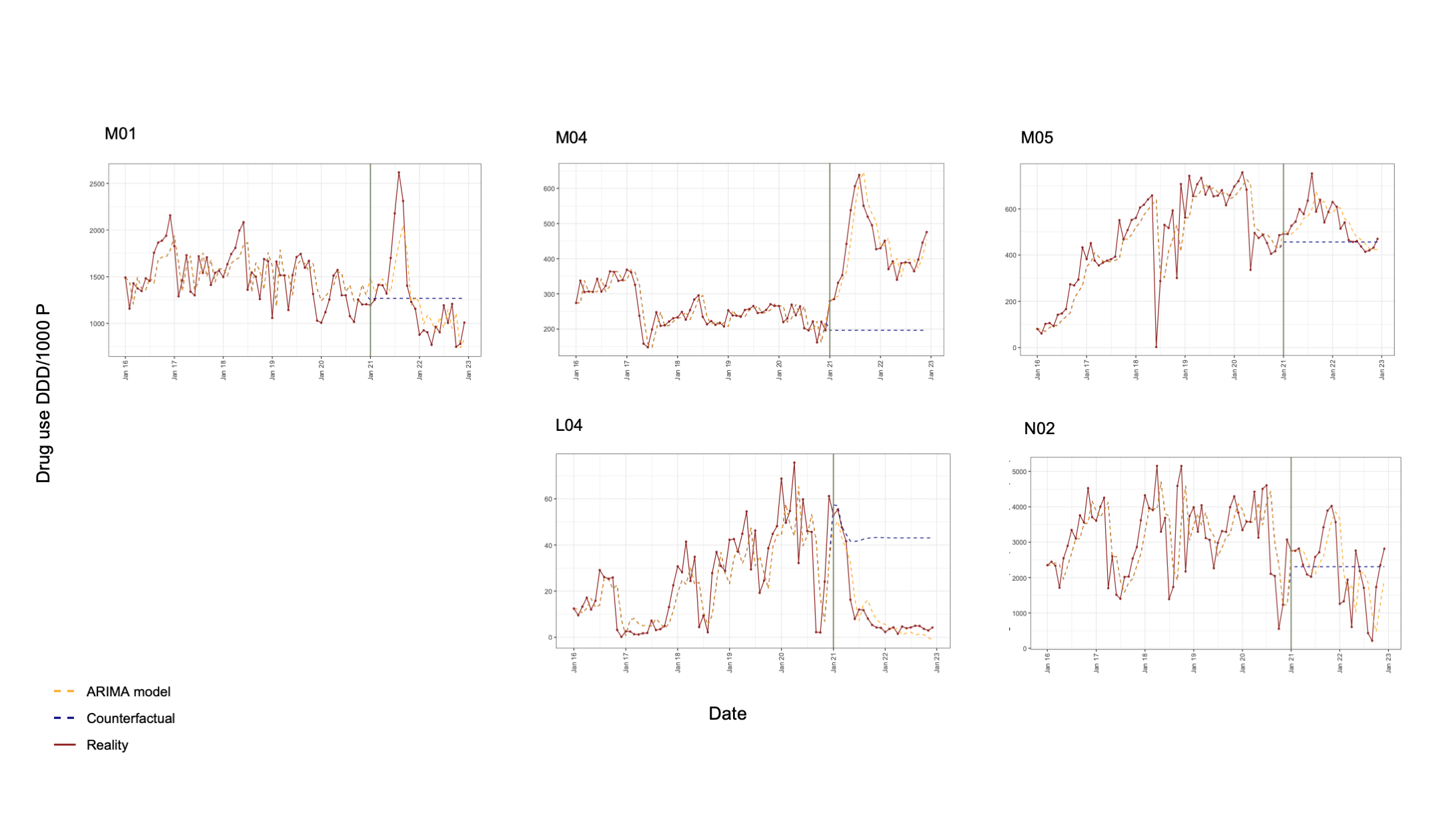

Supplement: S4 Fig — (TIF) [file pone.0297187.s004.tif]

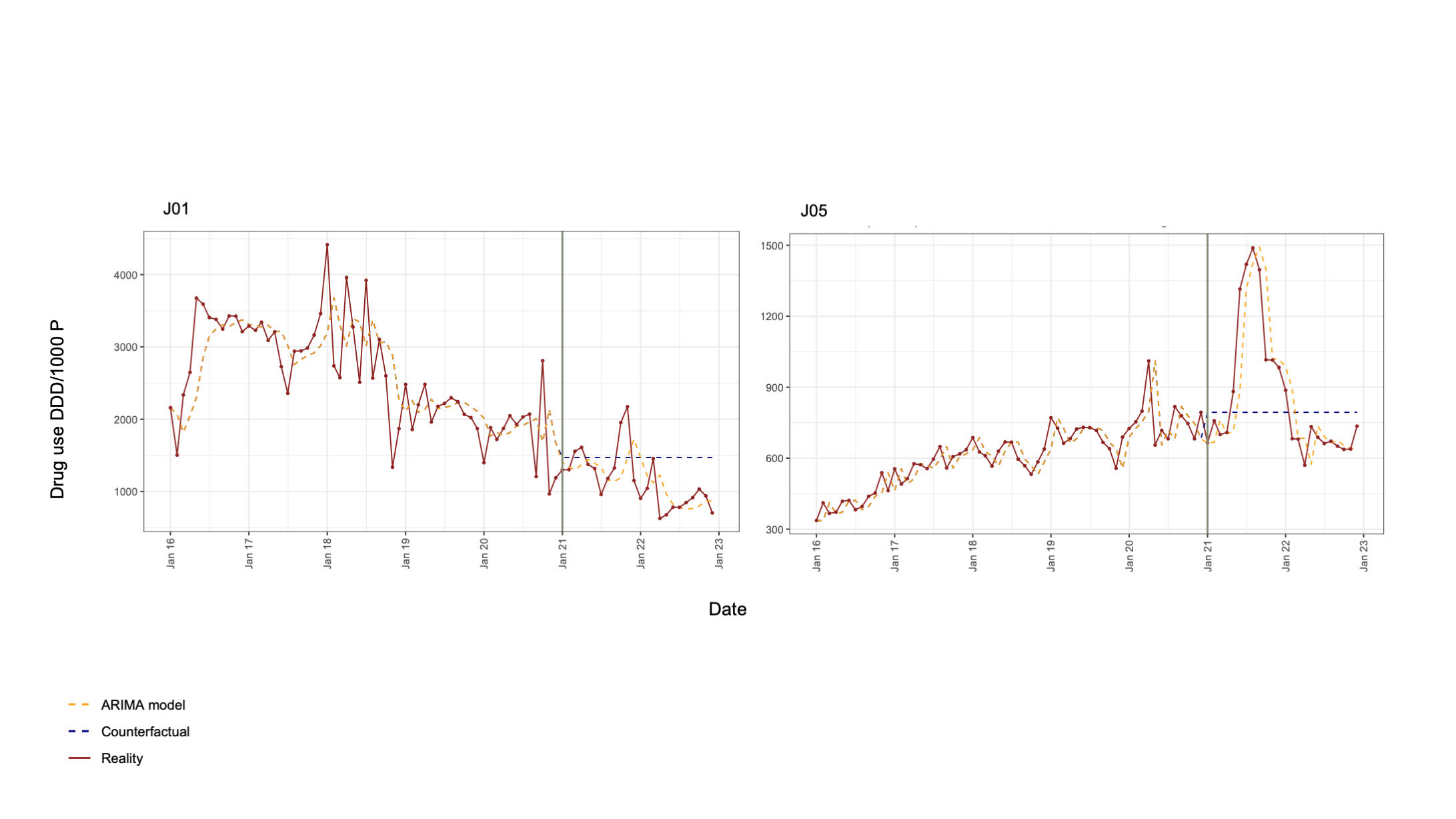

Supplement: S5 Fig — (TIF) [file pone.0297187.s005.tif]
