# Supplementary material for: Impact of COVID-19 on patterns of drug utilization: A case study at national hospital
Source: PLoS One. 2024 Jan 19;19(1):e0297187. doi: 10.1371/journal.pone.0297187 (PMC10798442; doi:10.1371/journal.pone.0297187)
Supplement: S1 Table — (DOCX) [file pone.0297187.s006.docx]

| **STT** | **Pharmacological group*** | **Number of prescriptions (%)** | |
| --- | --- | --- | --- |
|  |  | **Period 1** | **Period 2** |
| 1 | Analgesics, antipyretics; non-steroidal anti-inflammatory drugs (NSAIDs); and other drugs related to arthritis | 328,625 (26.27) | 89,385 (19.52) |
| 2 | Antiallergic medications used in cases of hypersensitivity | 130,445 (10.43) | 36,224 (7.91) |
| 3 | Detoxifying agents and drugs used in cases of poisoning | 14 (0.00) | 23 (0.01) |
| 4 | Anticonvulsant drugs, antiepileptic drugs | 70,795 (5.66) | 24,316 (5.31) |
| 5 | Antimicrobials | 220,226 (17.60) | 62,093 (13.56) |
| 6 | Medications for treating migraines | 12,328 (0.99) | 1,827 (0.40) |
| 7 | Anticancer drugs and immunomodulators | 2,448 (0.20) | 377 (0.08) |
| 8 | Medications for treating urinary tract diseases | 41,342 (3.30) | 27,976 (6.11) |
| 9 | Parkinson's disease medications | 8,575 (0.69) | 3,644 (0.80) |
| 10 | Hematological agents | 26,158 (2.09) | 5,588 (1.22) |
| 11 | Drugs active on cardiovascular system | 532,518 (42.57) | 232,894 (50.86) |
| 12 | Dermatological drugs | 23,703 (1.89) | 3,975 (0.87) |
| 13 | Disinfectants and antiseptics | 2 (0.00) | 0 (0.00) |
| 14 | Diuretic medications | 26,702 (2.13) | 11,932 (2.61) |
| 15 | Drugs active on digestive system | 359,585 (28.74) | 119,013 (25.99) |
| 16 | Drugs active on hormonal system | 276,310 (22.09) | 119,187 (26.03) |
| 17 | Muscle relaxants and cholinesterase inhibitors. | 85,007 (6.80) | 16,933 (3.70) |
| 18 | Medications for treating eye, ear, nose, and throat disorders | 144,525 (11.55) | 52,995 (11.57) |
| 19 | Psychotropic medications | 178,919 (14.30) | 58,574 (12.79) |
| 20 | Respiratory system medications | 76,642 (6.13) | 26,403 (5.77) |
| 21 | Fluid and electrolyte balance regulators, acid-base balance solutions, and other intravenous solutions | 6,751 (0.54) | 1,236 (0.27) |
| 22 | Minerals and vitamins | 204,749 (16.37) | 55,823 (12.19) |

**S1 Table. Number of prescriptions by pharmacological group**.

** The pharmacological group was classified based on Vietnamese Ministry of Health official legislation.*
