# Supplementary material for: Impact of COVID-19 on patterns of drug utilization: A case study at national hospital
Source: PLoS One. 2024 Jan 19;19(1):e0297187. doi: 10.1371/journal.pone.0297187 (PMC10798442; doi:10.1371/journal.pone.0297187)
Supplement: S2 Table — (DOCX) [file pone.0297187.s007.docx]

**S2 Table. Drug consumptions categorize by ATC code level 03 (DDD/1000P).**

| **ATC code level 03** | **Chemical, pharmacological, or therapeutic subgroup** | **DDD/1000P** | |
| --- | --- | --- | --- |
|  |  | **Period 1** | **Period 2** |
| **ALIMENTARY TRACT AND METABOLISM** | | | |
| A02 | Drugs for acid related disorders | 12691.90 | 15544.25 |
| A03 | Drugs for functional gastrointestinal | 560.05 | 306.03 |
| A04 | Antiemetics and antinauseants | 0.00 | 0.08 |
| A05 | Bile and liver therapy | 27.08 | 27.26 |
| A06 | Drugs for constipation | 250.12 | 287.51 |
| A07 | Antidiarrheals, intestinal anti-inflammatory/anti-infective agents | 815.37 | 871.42 |
| A09 | Digestives, incl. enzymes | 144.71 | 479.51 |
| A10 | Drugs used in diabetes | 68237.40 | 265337.58 |
| A11 | Vitamins | 1858.26 | 1095.67 |
| A12 | Mineral supplements | 1595.67 | 340.41 |
| A16 | Other alimentary tract and metabolism products | 44.23 | 26.58 |
| **BLOOD AND BLOOD FORMING ORGANS** | | | |
| B01 | Antithrombotic agents | 4738.64 | 8355.42 |
| B02 | Antihemorrhagics | 5.97 | 0.61 |
| B03 | Antianemic preparations | 575.00 | 539.50 |
| **CARDIOVASCULAR SYSTEM** | | | |
| C01 | Cardiac therapy | 2533.55 | 4313.84 |
| C02 | Antihypertensives | 47.75 | 105.89 |
| C03 | Diuretics | 397.56 | 559.29 |
| C04 | Peripheral vasodilatiors | 4.59 | 0.00 |
| C07 | Beta blocking agents | 1584.40 | 2982.72 |
| C08 | Calcium channel blockers | 3971.51 | 4506.15 |
| C09 | Agents acting on the renin-angiotensin system | 8626.02 | 14690.43 |
| C10 | Lipid modifying agents | 29319.98 | 51955.75 |
| **DERMATOLOGICALS** | | | |
| D01 | Antifungals for dermatological use | 14.83 | 37.83 |
| D10 | Anti-acne preparations | 0.29 | 0.00 |
| **GENITO URINARY SYSTEM AND SEX-HORMONES** | | | |
| G01 | Gynecological antiinfectives and antiseptics | 2.37 | 3.31 |
| G03 | Sex hormones and modulators of the genital system | 1.03 | 0.00 |
| G04 | Urologicals | 1016.05 | 3121.48 |
| **SYSTEMIC HORMONAL PREPARATIONS, EXCL. SEX HORMONES AND INSULINS** | | | |
| H01 | Pituitary and hypothalamic hormones and analogues | 0.40 | 22.18 |
| H02 | Corticosteroids for systemic use | 615.68 | 479.53 |
| H03 | Thyroid therapy | 238.05 | 251.65 |
| **ANTIINFECTIVES FOR SYSTEMIC USE** | | | |
| J01 | Antibacterials for systemic use | 2521.41 | 1142.61 |
| J02 | Antimycotics for systemic use | 5.21 | 2.32 |
| J04 | Antimycobacterials | 70.79 | 0.26 |
| J05 | Antivirals for systemic use | 616.10 | 775.05 |
| **ANTINEOPLASTIC AND IMMUNOMODULATING AGENTS** | | | |
| L04 | Immunosuppressants | 26.45 | 16.07 |
| **MUSCULO-SKELETAL SYSTEM** | | | |
| M01 | Antiinflammatory and antirheumatic products | 1491.81 | 1166.80 |
| M03 | Muscle relaxants | 587.63 | 391.45 |
| M04 | Antigout preparations | 255.01 | 400.93 |
| M05 | Drugs for treatment of bone diseases | 462.82 | 507.01 |
| **NERVOUS SYSTEM** | | | |
| N02 | Analgesics | 3127.21 | 2224.17 |
| N03 | Antiepileptics | 313.45 | 384.20 |
| N04 | Anti-parkinson drugs | 107.31 | 173.78 |
| N05 | Psycholeptics | 243.46 | 335.22 |
| N06 | Psychoanaleptics | 1132.45 | 1313.04 |
| N07 | Other nervous system drugs | 1403.60 | 1663.93 |
| **ANTIPARASITIC PRODUCTS, INSECTICIDES AND REPELLENTS** | | | |
| P01 | Antiprotozoals | 13.25 | 6.19 |
| P02 | Anthelmintics | 11.93 | 2.92 |
| **RESPIRATORY SYSTEM** | | | |
| R01 | Nasal preparations | 72.44 | 23.40 |
| R02 | Throat preparations | 1315.99 | 405.89 |
| R03 | Drugs for obstructive airway diseases | 2789.36 | 182.91 |
| R05 | Cough and cold preparations | 323.27 | 349.11 |
| R06 | Antihistamines for systemic use | 1346.59 | 1119.93 |
| **SENSORY ORGANS** | | | |
| S01 | Ophthalmologicals | 3.20 | 3.07 |
| **VARIOUS** | | | |
| V03 | All other therapeutic products | 0.09 | 0.04 |
