# Supplementary material for: Impact of COVID-19 on patterns of drug utilization: A case study at national hospital
Source: PLoS One. 2024 Jan 19;19(1):e0297187. doi: 10.1371/journal.pone.0297187 (PMC10798442; doi:10.1371/journal.pone.0297187)
Supplement: S3 Table — (DOCX) [file pone.0297187.s008.docx]

**S3 Table. The number of prescriptions’ data.**

| **Date** | **Real data** | **ARIMA model** | **Forecasting** |
| --- | --- | --- | --- |
| 01/01/2016 | 16821 | 16804.18 | 16804.18 |
| 01/02/2016 | 16758 | 16816.64 | 16816.64 |
| 01/03/2016 | 17185 | 16791.80 | 16791.80 |
| 01/04/2016 | 17325 | 17123.81 | 17123.81 |
| 01/05/2016 | 17236 | 17379.70 | 17379.70 |
| 01/06/2016 | 17086 | 17252.69 | 17252.69 |
| 01/07/2016 | 16900 | 16957.26 | 16957.26 |
| 01/08/2016 | 16831 | 16864.71 | 16864.71 |
| 01/09/2016 | 16739 | 16839.79 | 16839.79 |
| 01/10/2016 | 16718 | 16796.95 | 16796.95 |
| 01/11/2016 | 16648 | 16758.62 | 16758.62 |
| 01/12/2016 | 16475 | 16681.61 | 16681.61 |
| 01/01/2017 | 16176 | 16511.57 | 16511.57 |
| 01/02/2017 | 16471 | 16193.69 | 16193.69 |
| 01/03/2017 | 16818 | 16400.40 | 16400.40 |
| 01/04/2017 | 18205 | 16910.79 | 16910.79 |
| 01/05/2017 | 19412 | 18134.39 | 18134.39 |
| 01/06/2017 | 19220 | 19427.22 | 19427.22 |
| 01/07/2017 | 18045 | 19274.54 | 19274.54 |
| 01/08/2017 | 17576 | 17669.00 | 17669.00 |
| 01/09/2017 | 17734 | 17076.90 | 17076.90 |
| 01/10/2017 | 17625 | 17781.67 | 17781.67 |
| 01/11/2017 | 17634 | 18043.20 | 18043.20 |
| 01/12/2017 | 17592 | 17720.31 | 17720.31 |
| 01/01/2018 | 17501 | 17568.09 | 17568.09 |
| 01/02/2018 | 17565 | 17533.65 | 17533.65 |
| 01/03/2018 | 17677 | 17537.84 | 17537.84 |
| 01/04/2018 | 17860 | 17695.73 | 17695.73 |
| 01/05/2018 | 18073 | 17876.65 | 17876.65 |
| 01/06/2018 | 18441 | 18053.73 | 18053.73 |
| 01/07/2018 | 18952 | 18385.85 | 18385.85 |
| 01/08/2018 | 19437 | 18887.93 | 18887.93 |
| 01/09/2018 | 18598 | 19383.84 | 19383.84 |
| 01/10/2018 | 18484 | 18670.98 | 18670.98 |
| 01/11/2018 | 27787 | 18127.97 | 18127.97 |
| 01/12/2018 | 17964 | 26390.04 | 26390.04 |
| 01/01/2019 | 17407 | 21637.62 | 21637.62 |
| 01/02/2019 | 18161 | 14417.16 | 14417.16 |
| 01/03/2019 | 18358 | 16404.12 | 16404.12 |
| 01/04/2019 | 18048 | 21227.64 | 21227.64 |
| 01/05/2019 | 21489 | 18209.71 | 18209.71 |
| 01/06/2019 | 19260 | 20705.24 | 20705.24 |
| 01/07/2019 | 17538 | 20307.90 | 20307.90 |
| 01/08/2019 | 17966 | 17028.09 | 17028.09 |
| 01/09/2019 | 18238 | 16819.66 | 16819.66 |
| 01/10/2019 | 18394 | 19076.42 | 19076.42 |
| 01/11/2019 | 18455 | 18856.45 | 18856.45 |
| 01/12/2019 | 18219 | 18335.94 | 18335.94 |
| 01/01/2020 | 17919 | 18175.89 | 18175.89 |
| 01/02/2020 | 17878 | 17861.43 | 17861.43 |
| 01/03/2020 | 18074 | 17825.84 | 17825.84 |
| 01/04/2020 | 18083 | 18132.13 | 18132.13 |
| 01/05/2020 | 24939 | 18210.48 | 18210.48 |
| 01/06/2020 | 18893 | 23954.92 | 23954.92 |
| 01/07/2020 | 18828 | 21198.92 | 21198.92 |
| 01/08/2020 | 19201 | 16810.81 | 16810.81 |
| 01/09/2020 | 19025 | 17885.52 | 17885.52 |
| 01/10/2020 | 19475 | 20785.24 | 20785.24 |
| 01/11/2020 | 19430 | 19351.88 | 19351.88 |
| 01/12/2020 | 18886 | 19451.20 | 19451.20 |
| 01/01/2021 | 45053 | 38370.57 | 19182.14 |
| 01/02/2021 | 32182 | 43843.44 | 19218.20 |
| 01/03/2021 | 44656 | 35548.36 | 19204.58 |
| 01/04/2021 | 36048 | 39511.67 | 19188.35 |
| 01/05/2021 | 29696 | 39470.23 | 19149.21 |
| 01/06/2021 | 14895 | 30934.33 | 19184.30 |
| 01/07/2021 | 9261 | 13086.66 | 19188.51 |
| 01/08/2021 | 8835 | 9811.11 | 19180.80 |
| 01/09/2021 | 9714 | 10897.70 | 19179.20 |
| 01/10/2021 | 19478 | 14091.30 | 19177.74 |
| 01/11/2021 | 20825 | 19843.44 | 19181.38 |
| 01/12/2021 | 24184 | 22779.68 | 19181.35 |
| 01/01/2022 | 15750 | 22746.29 | 19180.06 |
| 01/02/2022 | 11492 | 14874.96 | 19180.08 |
| 01/03/2022 | 18607 | 9530.82 | 19180.22 |
| 01/04/2022 | 17831 | 16597.97 | 19180.51 |
| 01/05/2022 | 31082 | 22217.04 | 19180.43 |
| 01/06/2022 | 33535 | 29441.20 | 19180.28 |
| 01/07/2022 | 33346 | 34209.58 | 19180.32 |
| 01/08/2022 | 38131 | 32749.24 | 19180.35 |
| 01/09/2022 | 34354 | 33533.25 | 19180.37 |
| 01/10/2022 | 36541 | 35275.86 | 19180.35 |
| 01/11/2022 | 39347 | 34951.46 | 19180.34 |
| 01/12/2022 | 40101 | 38495.17 | 19180.34 |
