# Supplementary material for: Impact of COVID-19 on patterns of drug utilization: A case study at national hospital
Source: PLoS One. 2024 Jan 19;19(1):e0297187. doi: 10.1371/journal.pone.0297187 (PMC10798442; doi:10.1371/journal.pone.0297187)
Supplement: S4 Table — (DOCX) [file pone.0297187.s009.docx]

**S4 Table. Final fitted ARIMA Models provides the model specifications for the main ARIMA analyses presented in Table 2.**

| **Pharmacological group** | **ATC code level 03** | **Subgroup** | **Model** |
| --- | --- | --- | --- |
| All drugs |  |  | ARIMA(0, 1, 2) |
| Drugs active on cardiovascular system | C01 | Cardiac therapy | ARIMA(0, 1, 0) |
|  | C02 | Antihypertensives | ARIMA(1, 1, 1) |
|  | C03 | Diuretics | ARIMA(1, 1, 3) |
|  | C07 | Beta-blocking agents | ARIMA(1, 1, 3) |
|  | C08 | CCB | ARIMA(0, 1, 0) |
|  | C09 | RAASi | ARIMA(0, 1, 0) |
|  | C10 | Lipid modifying agents | ARIMA(0, 1, 1) |
|  | B01 | Antiplateles | ARIMA(0, 1, 0) |
| Drugs active on digestive system | A02 | Drugs for acid related disorders | ARIMA(0, 1, 1) |
|  | A03 | Drugs for functional gastrointestinal disorders | ARIMA(0, 1, 1) |
| Analgesics, antipyretics; non-steroidal anti-inflammatory drugs and other drugs related to arthritis | L04 | Immunosupressants | ARIMA(3, 1, 1) |
|  | M01 | Antiinflammatory and antirheumatic products | ARIMA(0, 1, 3) |
|  | M04 | Antigout preparations | ARIMA(0, 1, 0) |
|  | M05 | Drugs for treatment of bone diseases | ARIMA(0, 1, 1) |
|  | N02 | Analgesics | ARIMA(0, 1, 1) |
| Hormon and drugs active on endocrine system | A10 | Drugs used in diabetics | ARIMA(0, 1, 0) |
|  | H02 | Corticosteroids for systemic use | ARIMA(1, 1, 1) |
|  | H03 | Thyroid therapy | ARIMA(1, 1, 1) |
| Antimicrobials | J01 | Antibacterials for systemic use | ARIMA(0, 1, 1) |
|  | J05 | Antivirals for systemic use | ARIMA(0, 1, 0) |

******The ARIMA models were presented as ARIMA(p, d, q), in which: p is the number of autoregressive terms; d is the number of nonseasonal differences needed for stationarity, and q is the number of lagged forecast errors in the prediction equation.*
