# Supplementary material for: Impact of COVID-19 on patterns of drug utilization: A case study at national hospital
Source: PLoS One. 2024 Jan 19;19(1):e0297187. doi: 10.1371/journal.pone.0297187 (PMC10798442; doi:10.1371/journal.pone.0297187)
Supplement: Attachment — Submitted filename: PONE-D-23-22693.docx [file pone.0297187.s010.docx]

Review of PONE-D-23-22693: “Impact of COVID-19 on patterns of drug utilization: a case study at a national hospital”

Thank you for the opportunity to read this paper. In this paper, the authors employ data on the prescriptions dispensed from a national geriatric hospital in Ho Chi Minh City, Viet Nam to evaluate how the Covid-19 pandemic and associated policy responses impacted drug utilization. While other papers have evaluated this question in other countries, this is the first paper of which I am aware to consider the question in Viet Nam. While “different country” is sometimes not thought sufficient to warrant publication, different social and political responses to the pandemic warrant country-level analysis to inform public health response to future pandemics. Therefore, I feel the authors are evaluating a critically important question not just for Viet Nam, but for public health officials worldwide who can learn from the different responses that countries took.

The authors find that there was a significant surge in the number of prescriptions dispensed and the quantity of drugs dispensed. There was some heterogeneity in the types of drugs that saw such a surge; overall, the patterns were consistent with “stocking up” and didn’t drive long term increases in utilization.

By way of qualifications, I am familiar with this context, literature, and research methodology.

The authors investigate a crucially important question. As the manuscript currently stands, there are some aspects that could be strengthened to provide a more comprehensive assessment of the situation in Viet Nam and to give readers more confidence in the results. I hope that my comments are helpful in revising the manuscript.

**Major Comments:**

1) **Discussion of Covid-19 and Response in Viet Nam**

While there would certainly be domestic interest in the paper, I see the main value of the paper to readers outside Viet Nam, considering this paper alongside others that evaluate the response in other countries. Additionally, as we get further removed from the time period, exactly what happened when becomes a bit hazy. I recommend presenting a short discussion of the timeline of the COVID-19 pandemic in Viet Nam, Ho Chi Minh City, or both (whatever is most relevant). A graph showing the number of cases and deaths, along with annotations for the policy responses (e.g., lockdowns) would give the reader important context, especially since the timeline in Vietnam was a bit different than in other countries.

2) **Selection of Time Period Cut off**

Related to the comment above, my biggest methodological comment pertains to the selection of the Jan 2021 cutoff between Period 1 and Period 2. Is the rationale that this will completely capture the time period that began in Feb 2021 when the policy allowing 3-month supplies went into effect? I don’t disagree with this cutoff (though if it went into effect in Feb 2021, I might use Feb 2021 when the treatment started instead of Jan 2021). I think spelling this out more clearly and giving a rational for not using a later cutoff (such as the social distancing implementation in Jun or the April spike in cases) is important. My thought is that since the spike we actually see doesn’t occur until later on—Jun-Aug for most drugs—it is not being *caused* by the change in policy allowing refills…it’s something else causing it or we would see the spike in Feb/Mar. The drug refill policy might be a *precursor* but doesn’t seem to be what is driving the change. In this case, the effects of the change might be more clearly illustrated by using a later cutoff (e.g., when social distancing went into effect).

3) **Use of ARIMA for hypothesis testing**

ARIMA is commonly used in the pharmaceutical industry for predicting demand of medications and at least one reference [15] included an ARIMA analysis to show how COVID shifted utilization patterns, but its use in the academic literature for hypothesis testing is less well-known. I am not certain that most of the target audience for this paper will have had much exposure to ARIMA models since they are relatively uncommon in the medical literature. I would recommend presenting a short primer on the method including the functional form of the equation being estimated so that readers will have some context for the values presented in Table 2 (Change in level and Change in Slope).

4) **Dependent Variable**

I’m not sure that it’s fair to refer to the dependent variable as defined daily dose per 1000 inhabitants, unless the analysis is actually considering inhabitants (e.g., of a hospital catchment area). I think it’s fine to refer to defined daily dose per 1000 prescriptions; perhaps just say something like “we adapt the commonly used drug utilization metric…” As presented, what we are seeing is the average DDD per prescription increased (i.e., people got larger drug supplies than they did in the past). But this really only tells half the story.

I strongly recommend presenting additional analyses using a dependent variable that represents the number of prescriptions filled, and perhaps the total amount of drug dispensed so that readers can understand the complete picture as it pertains to drug access. From the perspective of a pharmaceutical supply chain manager building a buffer to allow for a pandemic, if the DDD/1000P spiked as shown but the *number of prescriptions* declined, the total amount of drug needed could stay the same or even go down. Similarly, it could be the case that two things are true simultaneously—people who filled prescriptions stocked up but there were many people unable to access their medications (Ref [15] showed this happened elsewhere in the world, I think).

**Minor Comments**

*Figure 1:* I don’t really understand the bottom section of this figure—the analysis seems to refer to the portion on the left hand said (1,060,507 in Period 1 and 644,944 in Period 2). Where does the 6,051,509 number come from? Recommend explaining this NT1 and NT2 and NT3 notation. On the left hand side, there is an n1, n3, and n4 but no n2. I found this notation a bit confusing.

*Study setting and design:* There is a comment “During the fourth wave of COVID-19, Thong Nhat hospital was in charge of a 1000 bed multi-class COVID-19 field hospital.” I wasn’t sure how this related to the study. Obviously, this was important, difficult, and valuable work (and I’m guessing, but it wouldn’t surprise me to learn that one or more of the authors personally contributed to this effort). Is this implication that because of this strain on resources, there was a need to reduce the number of non-emergent patients visiting the hospital (e.g., for chronic medication refills) leading to the 90-day supply exception discussed in the next paragraph? If so, I recommend just saying this directly.

*Note on Table 1:* The note reads “We presented the commonly variables, please prefer in the Supplementary…” Recommend checking grammar and rewording to make it more clear.

*Viet Nam* vs *Vietnam*: The manuscript uses both “Viet Nam” and “Vietnam” as the transliterated country name. Recommend sticking with one spelling for consistency (“Viet Nam” appears more frequently in the manuscript).
