# Supplementary material for: Impact of COVID-19 on patterns of drug utilization: A case study at national hospital
Source: PLoS One. 2024 Jan 19;19(1):e0297187. doi: 10.1371/journal.pone.0297187 (PMC10798442; doi:10.1371/journal.pone.0297187)
Supplement: Attachment — Submitted filename: Response to reviewers.docx [file pone.0297187.s011.docx]

**Reviewer 1 comments:**

**1)** Discussion of Covid-19 and Response in Viet Nam

While there would certainly be domestic interest in the paper, I see the main value of the paper to readers outside Viet Nam, considering this paper alongside others that evaluate the response in other countries. Additionally, as we get further removed from the time period, exactly what happened when becomes a bit hazy. I recommend presenting a short discussion of the timeline of the COVID-19 pandemic in Viet Nam, Ho Chi Minh City, or both (whatever is most relevant). A graph showing the number of cases and deaths, along with annotations for the policy responses (e.g., lockdowns) would give the reader important context, especially since the timeline in Vietnam was a bit different than in other countries.

***Response:*** We appreciate your consideration of our findings to international readers. We strongly agree with you to include the timeline of COVID-19 pandemic in Ho Chi Minh City for readers to easily follow the study design and analysis. We have encompassed a new figure (Figure 1) to illustrate the whole timeline and each timepoint in detail. Unfortunately, the database related to COVID-19 cases and deaths for the whole pandemic periods (from 2020 to 2022) is not publicly accessed. The ownership of the data, which is the Viet Nam Ministry of Health, permits to view monthly summarize data only. Therefore, we are unable to provide the detailed monthly situation about the pandemic in Ho Chi Minh City within the whole periods. However, we have presented it through aggregating official, important news during the occurrence of COVID-19 in Ho Chi Minh City, which comprehensively decribe the pandemic context in the city. Please refer to Figure 1 for the description. We are open to any further amendment if required.

**2)** Selection of Time Period Cut off

Related to the comment above, my biggest methodological comment pertains to the selection of the Jan 2021 cutoff between Period 1 and Period 2. Is the rationale that this will completely capture the time period that began in Feb 2021 when the policy allowing 3-month supplies went into effect? I don’t disagree with this cutoff (though if it went into effect in Feb 2021, I might use Feb 2021 when the treatment started instead of Jan 2021). I think spelling this out more clearly and giving a rational for not using a later cutoff (such as the social distancing implementation in Jun or the April spike in cases) is important. My thought is that since the spike we actually see doesn’t occur until later on—Jun-Aug for most drugs—it is not being *caused* by the change in policy allowing refills…it’s something else causing it or we would see the spike in Feb/Mar. The drug refill policy might be a *precursor* but doesn’t seem to be what is driving the change. In this case, the effects of the change might be more clearly illustrated by using a later cutoff (e.g., when social distancing went into effect).

***Response:*** You have raised a critical question. Regarding the context of pandemic timeline in Ho Chi Minh City, the first COVID-19 case was found by the end of 2019. The government effectively controlled the pandemic by implementing “quarantine policy” or “zero-COVID policy” to ensure there was no COVID-19 infected case in community. Which means that patients who had COVID-19 positive test (rapid test not a confirmed one) must be quarantined in COVID-19 related healthcare facilities, such as hospitals or COVID-19 field hospitals.

There was no COVID-19 case in community was recorded and announced by 31^st^ December 2020. However, the COVID-19 spread was still occurring in Ho Chi Minh City during this time, mainly due to cross-infectious cases were qurantined in the COVID-19 related fallacies.

The subsequent COVID-19 community case was found on 29^th^ January 2021 together with the complicated situations of pandemic in other countries could raise concerns in outpatient requiring long-term drug use. Therefore, it could change the drug use in our hypothesis. Our study concentrated on **the effect of COVID-19** (minimizing the influence of mitigated policies). We supposed that choosing January 2021 as an intervention timepoint is appropriate since it was the time when COVID-19 started to rise again, and the time when Ho Chi Minh City was catastrophically affected by the pandemic indeed. Besides, if we used a cutoff on Jun-Aug, the data could be significantly influenced by a very strict social distancing (which we want to avoid since our study mainly focused on the early stage and the effect of COVID-19 only).

**3)** Use of ARIMA for hypothesis testing

ARIMA is commonly used in the pharmaceutical industry for predicting demand of medications and at least one reference [15] included an ARIMA analysis to show how COVID shifted utilization patterns, but its use in the academic literature for hypothesis testing is less well-known. I am not certain that most of the target audience for this paper will have had much exposure to ARIMA models since they are relatively uncommon in the medical literature. I would recommend presenting a short primer on the method including the functional form of the equation being estimated so that readers will have some context for the values presented in Table 2 (Change in level and Change in Slope).

***Response:*** We have added comments about the advantages of using ARIMA in ITS design and definitions for two variables “Step change” and “Ramp”.

**4)** Dependent Variable

I’m not sure that it’s fair to refer to the dependent variable as defined daily dose per 1000 inhabitants, unless the analysis is actually considering inhabitants (e.g., of a hospital catchment area). I think it’s fine to refer to defined daily dose per 1000 prescriptions; perhaps just say something like “we adapt the commonly used drug utilization metric…” As presented, what we are seeing is the average DDD per prescription increased (i.e., people got larger drug supplies than they did in the past). But this really only tells half the story.

I strongly recommend presenting additional analyses using a dependent variable that represents the number of prescriptions filled, and perhaps the total amount of drug dispensed so that readers can understand the complete picture as it pertains to drug access. From the perspective of a pharmaceutical supply chain manager building a buffer to allow for a pandemic, if the DDD/1000P spiked as shown but the *number of prescriptions* declined, the total amount of drug needed could stay the same or even go down. Similarly, it could be the case that two things are true simultaneously—people who filled prescriptions stocked up but there were many people unable to access their medications (Ref [15] showed this happened elsewhere in the world, I think).

***Response:*** Regarding using the number of prescriptions filled as an additional dependent variable, we acknowledge that presenting it would fully provide evidence to strengthen and futher discuss on our outcomes. Unfortunately, due to the limitation in linking outpatient’s personal information which we have clarified in our study’s limitation, the study could only collect an individual ID for each prescription. As there were no variables having the “repetitiveness”, the study was unable to identify which individuals followed the filled prescription.

In our study, we have examined the change of *the number of prescriptions* affected by the COVID-19 intervention, which resulted in an immediately significant surge (step change) by 19,425 prescriptions (p < 0.001). With the formular to calculate DDD/1000P is $\frac{1000\times DDD}{number of prescriptions (monthly)}$, we can deduce that if the DDD/1000P of a drug specific group increased, DDD would increase. To deduce if DDD/1000P decreased, we have added a Table in Supplementary providing the monthly data of the number of prescriptions (real data, ARIMA data, and forecast data); therefore, we believe that DDD (the amount of drug dispensed) could clearly deduce from the formula mentioned above. We hope that this complement is sufficient to address your concern.

**5)** *Figure 1:* I don’t really understand the bottom section of this figure—the analysis seems to refer to the portion on the left hand said (1,060,507 in Period 1 and 644,944 in Period 2). Where does the 6,051,509 number come from? Recommend explaining this NT1 and NT2 and NT3 notation. On the left hand side, there is an n1, n3, and n4 but no n2. I found this notation a bit confusing.

***Response:*** We agree that there are some typos in notations, thank you for pointing it out.

The number 6,051,509 means the number of drugs calculated from total prescriptions satisfied selection criteria. For example, there are total two prescriptions collected: Prescription 1 has 3 drugs prescribed, and prescription 2 has 5 drugs prescribed, so 8 is the total number of prescription drugs. We will add an annotation under the figure to make it clearer.

***6)*** *Study setting and design:* There is a comment “During the fourth wave of COVID-19, Thong Nhat hospital was in charge of a 1000 bed multi-class COVID-19 field hospital.” I wasn’t sure how this related to the study. Obviously, this was important, difficult, and valuable work (and I’m guessing, but it wouldn’t surprise me to learn that one or more of the authors personally contributed to this effort). Is this implication that because of this strain on resources, there was a need to reduce the number of non-emergent patients visiting the hospital (e.g., for chronic medication refills) leading to the 90-day supply exception discussed in the next paragraph? If so, I recommend just saying this directly.

***Response:*** We admitted that this sentence did not clearly convey the context. The field hospital was established in order to gather and provide treatment for all patients who had diverse spectrums of COVID-19 symptoms (focusing on COVID-19 treatment). This comment was added to explain the reason why our data encompass only a minority of COVID-19 patients even though a surge of new COVID-19 cases was noted during the fourth wave of COVID-19 (if a patient visited the hospital suspected that they had COVID-19 symptoms, the hospital would note the diagnosis of COVID-19 in the prescription for outpatient, then transferred the patient to the field hospital). We have added additional comments to make it clearer.

***7)*** *Note on Table 1:* The note reads “We presented the commonly variables, please prefer in the Supplementary…” Recommend checking grammar and rewording to make it more clear.

***Response:*** We have changed the note on Table 1 into “We presented the common variables, please refer to the Supplementary for full results of the number of prescriptions by pharmacological group”.

***8)*** *Viet Nam* vs *Vietnam*: The manuscript uses both “Viet Nam” and “Vietnam” as the transliterated country name. Recommend sticking with one spelling for consistency (“Viet Nam” appears more frequently in the manuscript).

***Response:*** Thank you for your recommendation. We have fixed to “Viet Nam” in the manuscript.

**Reviewer 2 comments:**

**1)** In the results section of the abstract, authors state “The median age of the patients ranged from 58 to 67 years old”. Do they mean the median in period 1 was 58 and and 67 in period 2? Authors could clarify this as median is one value and not a range. Authors could provide the interquartile range for each median value.

***Response:*** Thank you for pointing it out. We have fixed the sentence presented median values and added interquartile ranges.

**2)** Authors could use the full name of the month e.g., December instead of Dec for consistency throughout the document.

***Response:*** We have fixed the name of the month for the consistency.

**3)** Could the authors provide a table or figure of the timeline of the interventions/strategies implemented by the government/ministry of health to reduce the spread of COVID-19?

***Response:*** We have included an additional figure of the timeline of control measures implemented in the manuscript. Thank you for your suggestion.

**4)** Is there a justification or reason for excluding those aged below 18?

***Response:*** Our study use DDD/1000P (Defined daily dose per 1000 prescriptions) as the main dependent variable for evaluating drug use. According to the requirement of calculating DDD, which cannot apply for patients under 18 years old, we decided to exclude prescriptions aged below 18.

**5)** Could the authors add on Table 1 the missing data for each characteristic to help the reader know what proportion of data was missing for each characteristic as I understand the analysis was done for complete data a.k.a Complete Case Analysis (CCA).

***Response:***

**6)** Were any of the patient characteristics such as age, comorbidities etc accounted for in the interrupted time series model?

***Response:*** The patient characteristics in Table 1 did not participate in the model. However, we encompassed it in order to provide readers the general description of the population in the hospital chosen for our study, which concentrated on elderly patients with chronic diseases.

**7)** Authors used ARIMA model to fit the ITS models, could Autocorrelation and partial autocorrelation function (ACF and PACF) plots be provided in the supplementary materials.

***Response:*** There are 2 ways to determine the parameters for ARIMA model: manual or automatic modelling using auto.arima( ) in R. The process can be found in: **Forecasting Principles and Practice** by *Rob J Hyndman and Geogre Athanasopoulos*. To simplify, our study performs automatic modelling to determine the best fitting ARIMA model. This automatic process does not require the output of ACF and PACF plots. After receiving ARIMA parameters from running auto.arima( ) function, we plotted the data from ARIMA model and reality data to examine whether two datasets were fitted, thus ensuring that ARIMA model was appropriate.

**8)** Authors could reference the R packages and also the R software used for the ARIMA model if possible as it’s good practice to recognize these softwares.

***Response:*** Our study data was analyzed referencing “**Interrupted time series analysis using autoregressive integrated moving average (ARIMA) models: a guide for evaluating large-scale health interventions**” by *Schaffer, A.L., Dobbins, T.A. & Pearson, SA*; therefore, we recommend you refer this publication. They provide a complete R code for running the model and a simple example supporting it.

**9)** In the sentence “the average number of drugs per prescription and the drug consumptions, compared to their counterfactuals in order to assess.[22–24]”. Seems something is missing at the end of the sentence? In order to assess what?

***Response:*** We have removed “*in order to assess*” in the sentence. Full sentence after fixing is “We used the model to detect the immediate change in level (step change) and change in slop (ramp) monthly in the number of prescriptions, the average number of drugs per prescription and the drug consumptions compared to their counterfactuals”.

**10)** In the sentence. In the first period, the 30- to below 60-year-old was the highest prevalence. Do authors mean the majority of the patients were aged 30-60 in your study dataset? Maybe rewording of the sentence could make it clearer

***Response:*** We have changed the sentence into “In the first period, the percentage of patients aged from 30 to below 60 was the highest prevalence”.

**11)** Authors could define abbreviations the first time they are used in the manuscript

***Response:*** We have added abbreviations in the first of in the manuscript.

**12)** Authors could check for grammatical errors through the document.

***Response:*** We have fixed grammatical errors in the manuscript.
