# Supplementary material for: Impact of COVID-19 on patterns of drug utilization: A case study at national hospital
Source: PLoS One. 2024 Jan 19;19(1):e0297187. doi: 10.1371/journal.pone.0297187 (PMC10798442; doi:10.1371/journal.pone.0297187)
Supplement: Attachment — Submitted filename: Rebuttal letter.docx [file pone.0297187.s012.docx]

**Changes in references: no previous references were retracted. We solely added additional references with the following purposes**

**6 references were added in order to contextualize the COVID-19 circumstance in Ho Chi Minh City, Viet Nam**

**10.** Covid deaths rising in Ho Chi Minh City - VnExpress International [Internet]. [cited 2023 Sep 26]. Available from: <https://e.vnexpress.net/news/news/covid-deaths-rising-in-ho-chi-minh-city-4397493.htm>

**11.** Review of COVID-19 fight in HCMC [Internet]. [cited 2023 Sep 26]. Available from: <https://en.baochinhphu.vn/review-of-covid-19-fight-in-hcmc-11142209.htm>

**22.** HCMC confirms first coronavirus case linked to Hai Duong - VnExpress International [Internet]. [cited 2023 Sep 26]. Available from: https://e.vnexpress.net/news/news/hcmc-confirms-first-coronavirus-infection-linked-to-northern-hotspot-4228920.html

**23.** Tan Son Nhat airport staff among four confirmed Covid-19 community transmissions - VnExpress International [Internet]. [cited 2023 Sep 26]. Available from: https://e.vnexpress.net/news/news/tan-son-nhat-airport-staff-among-four-confirmed-covid-19-community-transmissions-4232669.html

**24.** HCM City orders temporary closure of non-essential services [Internet]. [cited 2023 Sep 26]. Available from: https://vietnamnews.vn/society/939308/hcm-city-orders-temporary-closure-of-non-essential-services.html

**25.** HCMC relaxes social distancing measures, gradually restarts economic activities [Internet]. [cited 2023 Sep 26]. Available from: <https://en.baochinhphu.vn/hcmc-relaxes-social-distancing-measures-gradually-restarts-economic-activities-11142370.htm>

**2 references supported the rationale of using ARIMA method for analyzing data**

**28.** Turner SL, Karahalios A, Forbes AB, Taljaard M, Grimshaw JM, McKenzie JE. Comparison of six statistical methods for interrupted time series studies: empirical evaluation of 190 published series. BMC Med Res Methodol. 2021 Jun 26;21(1):134.

**30.** Li L, Cuerden MS, Liu B, Shariff S, Jain AK, Mazumdar M. Three Statistical Approaches for Assessment of Intervention Effects: A Primer for Practitioners. Risk Manag Healthc Policy. 2021;14:757.

**Response to reviewers**

**Reviewer 1:**
I think the paper would benefit from some contextualization (maybe in the discussion section) to put some perspective around the changes…it looks like the changes in utilization were really driven by the patient population being served. This has important implications for the conclusion statements and key takeaways (e.g., “COVID-19 resulted in a sudden, non-significant increase in overall drug consumption levels among outpatients.”). It doesn’t look (to me) as if suddenly people needed more cardiovascular drugs…this result is driven by the patients who are still being served. I don’t think this compromises the results but it does add some important perspective—for the clinicians still serving the non-Covid-19 patients, their patients will-on average-be much sicker.

**Response:** We really appreciate that you pointed out the issue. After revising the paper, we think that it is crucial to complement information about the outpatients’ context in the hospital.

Regarding this hospital, most of patients claim their prescription with the social health insurance, and it can only be done in the registered hospital in the insurance. There was a large proportion of chronic diseases observed in the study population, which indicated that these outpatients have been examined in this hospital for a while. Besides, increases in the drug consumption in the period 2 were mostly driven by the surge of the consumption observed in the initial stage of the pandemic outbreak, which illustrated the stockpiling behavior, and thus supporting the abovementioned indication. Therefore, we can anticipate that there were few first-time outpatients with chronic diseases as the majority of patients has the long-term follow-up at the hospital. We believe that this is equivalent to “the patient population being served” in your opinion. We have added the comments with respect to the outpatients in the discussion.

**Reviewer 2:**

Could the authors add on Table 1 the missing data for each characteristic to help the reader
know what proportion of data was missing for each characteristic as I understand the analysis
was done for complete data a.k.a Complete Case Analysis (CCA).

**Response:** We sincerely apologize that this comment was missed out during the first revision. In terms of the first step of excluding missing data, missing data mainly encompassed the omitted values of age and diagnosed diseases. Therefore, we were unable to aggregate such missing values into each category of characteristics in the table. We think that missing data excluded did not affect the analysis of the finalized data.
